# Gravity Influences How We Expect a Cursor to Move

**DOI:** 10.1177/03010066211065229

**Published:** 2021-12-17

**Authors:** Eli Brenner, Milan Houben, Ties Schukking, Emily M. Crowe

**Affiliations:** Department of Human Movement Sciences, Faculty of Behavioural and Movement Science, 1190 Vrije Universiteit Amsterdam, The Netherlands

**Keywords:** frames of reference, pointing/hitting, cursor, computer mouse, gravity, visually guided movements

## Abstract

We expect a cursor to move upwards when we push our computer mouse away. Do we expect it to move upwards on the screen, upwards with respect to our body, or upwards with respect to gravity? To find out, we asked participants to perform a simple task that involved guiding a cursor with a mouse. It took participants that were sitting upright longer to reach targets with the cursor if the screen was tilted, so not only directions on the screen are relevant. Tilted participants’ performance was indistinguishable from that of upright participants when the screen was tilted slightly in the same direction. Thus, the screen's orientation with respect to both the body and gravity are relevant. Considering published estimates of the ocular counter-roll induced by head tilt, it is possible that participants actually expect the cursor to move in a certain direction on their retina.

People readily guide a cursor across a vertical screen by moving their computer mouse across a horizontal surface. Moving the mouse further away may so readily be associated with positions higher on the screen because things that are further away on a horizontal surface are generally higher in one's field of view. If so, performance might depend on the orientation of the screen with respect to the person's body or to gravity. We therefore asked 30 naive right-handed participants to move a cursor to consecutive targets as quickly as possible (as in Brenner et al., 2020) both when sitting upright and when tilted by 45° so that their body was no longer aligned with gravity (see images in Figure 1). The screen was also tilted by various extents with respect to the participant's body, and therefore also with respect to gravity.

**Figure 1.**
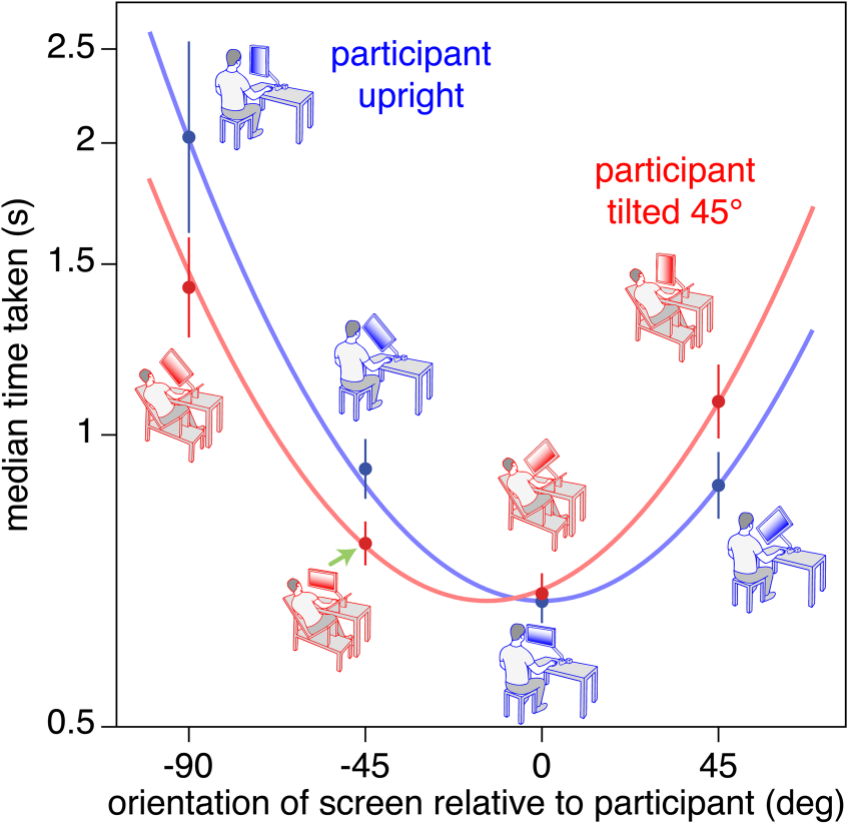
Participants either sat upright (blue drawings) or tilted 45° to the left on a specially designed chair with a surface supporting the left side of their body and surfaces for their feet and the mouse that were also tilted by 45° (red drawings). In each case the screen was tilted either −90°, −45°, 0°, or 45° with respect to the participant's body. Data points show the mean and 95% confidence intervals across the 27 participants’ median values for each condition. The green arrow illustrates the condition in which the participant was tilted but the screen was aligned with gravity (see screen orientation in drawing).

Participants were instructed not to rotate their head with respect to their body. They used a lightweight computer mouse to move a 7 mm diameter black disk (the cursor) to a 17 mm diameter blue disk (the target) on a 59.5 by 33.5 cm screen. Once the center of the cursor remained within the target for 50 ms a new target appeared, 16.7 cm from the previous one in a random direction. The participants’ task was to hit as many targets as possible within 75 s. There was one such session for each of the eight conditions, in random order. After finishing all eight conditions the participants were asked whether moving the mouse in the tilted conditions was uncomfortable. We excluded the data of three participants who answered that it was. The Scientific and Ethical Review Board of the Faculty of Behavioural and Movement Science approved the study.

We determined the median time each participant took to reach the target for each condition ( $t_c$ ), and show these times (on a logarithmic scale) as a function of the screen's orientation with respect to the participant ( $\alpha $ ). We also fit a parabolic function to each participant's data: $\log t_c = \log t_{min} + r\lpar \alpha + \beta \rpar ^2$ . The three fit parameters are the minimal time taken ( $t_{min}$ ), the rate at which time increases when the screen is rotated away from the orientation at which the time taken is minimal (*r*), and the extent to which gravity influences the optimal orientation when the participant is tilted ( $\beta $ ). There are two curves in the figure because $\beta $ is zero when the participant is upright (blue curve) and has the fit value when the participant is tilted (red curve). The plotted curves are based on the means of the participants’ fit values.

Performance was not systematically worse when the participant was tilted. Across participants, the mean fit values of $t_{min}$ and $\beta $ are 673 ms and 14° (with 95% confidence intervals of 42 ms and 4°). When the participant was upright, the median time taken to reach the target clearly increased as the screen was tilted, showing that participants did not only consider how the cursor moved across the screen. When the participant was tilted by 45° performance depended on the orientation of the screen in a similar manner as when the participant was upright, but the optimal angle of the screen was shifted by about 14° in the direction in which the participant was tilted. Thus, the direction in which the cursor moves with respect to the participant matters, but so does the direction with respect to gravity.

A possible explanation for the finding that participants are influenced by the cursor's direction of motion relative to gravity as well as to their body is that the arm is actually guided by information in retinal coordinates. We did not measure our participants’ eye movements, but studies that examined torsional eye orientations when the head is tilted relative to the body show that the eyes rotate in the opposite direction than the head (Lim et al., 2017; Schworm et al., 2002; Yamao et al., 2017). In those studies the eyes rotated 6–10° for a head tilt of 45°, but maybe the rotation is larger when the participant's whole body is tilted or when the participant is performing a visuo-motor task rather than simply fixating. Moreover, we may have overestimated the effect of gravity by a few degrees, because although participants did not visibly rotate their heads when seated in the tilted chair, when we checked the orientation of the head in photographs we did see a tendency to tilt the head towards the upright relative to the body by one or two degrees. Thus, when people move the mouse they may expect the cursor to move in its normal manner on the retina, with directions on the retina becoming slightly different due to ocular counter-roll when the head is tilted.
